# Great Challenges in Organic Chemistry

**DOI:** 10.3389/fchem.2017.00052

**Published:** 2017-07-12

**Authors:** Iwao Ojima

**Affiliations:** Department of Chemistry, Stony Brook University Stony Brook, NY, United States

**Keywords:** grand challenges, interface of organic chemistry, new frontiers, interdisciplinary, multidisciplinary

The current scope of “organic chemistry journals” typically covers the theory and practice of (i) new synthetic methods and methodologies, (ii) isolation and synthesis of natural products, (iii) organic reaction mechanisms based on physical and theoretical chemistry approaches, (iv) bioorganic and medicinal chemistry, (v) organometallic chemistry, (vi) molecular recognition and supramolecular chemistry, and (vii) polymers and materials chemistry.

These categories or branches have been established over years, reflecting the evolution of this field of chemistry on the basis of organic chemistry principles. The evolution will naturally continue in organic chemistry, which is based on clear understanding of the two- and three-dimensional chemical structures, as well as their relations to stability, reactivity, and other chemical properties. This characteristic feature of organic chemistry is very unique and unparalleled to any other disciplines in chemical sciences. Thus, the structure–property, structure–activity, and structure–function relationships of organic compounds will keep serving as core themes in organic chemistry research.

It is very clear that organic chemistry has been thriving by expanding its territories through exploration of the interfaces with other science disciplines. Thus, organic chemistry is undoubtedly serving as the core chemical science for the advancement of science and technology with clear goals to benefit human life and society.

Accordingly, one of the grand challenges in organic chemistry is *how to explore new frontiers at the interface of organic chemistry and other science or technology fields*. In the past, the majority of interdisciplinary research was between two disciplines in two different laboratories. But now it is necessary to take multidisciplinary approaches, involving multiple disciplines and laboratories, for tackling significant scientific or technological problems. Under these circumstances, organic chemists must evolve into open-minded researchers who can effectively communicate and collaborate with other researchers from different disciplines. In order to achieve this goal, organic chemists should have good knowledge of other disciplines to understand the whole picture of the project. Thus, another grand challenge for organic chemists is *how to evolve into a key player in a multidisciplinary research project by cultivating the ability to effectively communicate and collaborate with other project team members from different disciplines*. Then another closely related grand challenge is *how to cultivate the next generation of organic chemists who can survive and thrive in the broad interfaces of organic chemistry and other science/technology disciplines*. Since traditional organic chemists enjoyed research only in their own comfortable playgrounds, these will be great challenges in research and education that organic chemists must face.

Since “chemistry” has become the central core molecular science for energy, environment, sustainability, materials, biology, and medicine, great challenges in “organic chemistry” reflect more or less the same trend. In addition, advances in computing capacity and capabilities have opened avenues for big data treatment and analysis, systems chemistry, accurate simulations and predictions. Accordingly, it would be safe to say that *the great challenges and successes in organic chemistry would reside at the interface with energy, environment, sustainability, materials, biology, medicine, and computer science*.

Now, let's move on to the examples of great challenges in branches of organic chemistry.

At the interface with energy, solar energy and energy storage have been predominantly led by inorganic materials. Thus, there is a great challenge for organic chemists to create organic or hybrid materials to outperform existing inorganic materials.

At the interface with sustainability and environmental science, a challenge is the development of efficient chemical processes converting industrial and agricultural wastes, industrial bi-products, carbon dioxide, greenhouse gases such as fluoroform, recovered plastics, etc., to useful chemicals without producing another waste. If these processes include efficient photochemical processes utilizing solar energy, it will be ideal.

At the interface with materials, many great challenges can be envisioned, and already numerous research and development themes are ongoing in this field. The challenge here is how organic chemistry can play a key role in polymer and materials chemistry. The development of new, selective, and efficient polymerization methods and methodologies exploiting organometallic chemistry and organocatalysis make a huge impact on this endeavor. Supramolecular chemistry plays a significant role in the creation of novel organic, organometallic, coordination complex, and hybrid materials, wherein organic chemistry can make critical contributions. “Molecular machines” have already emerged as a new concept but how can organic chemists construct organic functional devices consisting of molecular machines with macroscopic motions?

At the interface with biology and medicine, there are plethora of great challenges for organic chemists, e.g., epigenerics, DNA damage and repair, gene editing, nanomedicine, nano-formulations, molecular imaging, drug discovery and development, antibody-drug conjugates, next-generation fluorescence dyes for super-resolution imaging of living cells, just to mention a few. Chemical biology has evolved from bioorganic chemistry and biochemistry which provides powerful tools to investigate biological problems at the molecular level. For drug discovery and pharmaceutical sciences, synthetic organic and medicinal chemistry are indeed essential. However, the challenge here is how next-generation organic/medicinal chemists can play a key role in the whole drug discovery process, i.e., not simply serve as a contract research organization (CRO) for preparing library of compounds in a classical medicinal chemistry manner. Next-generation organic/medicinal chemists should be able to fully engage in drug design based on structural and computational biology. Physical organic chemists should be able to apply kinetics and thermodynamics analysis, especially in combination with molecular imaging, for the accurate evaluation of drug efficacy and mode of action, and better drug design.

At the interface with computer science, there are numerous great challenges for organic chemistry. How can computational organic chemistry expand quantum mechanics analysis and prediction for organic reaction mechanism and catalytic cycles with increasing molecular sizes without X-ray crystal structures? How can computational organic chemists connect big data science with organic chemistry to explore “systems organic chemistry”? How can organic chemists and computational chemists work together to do rational design for new, selective, and efficient organic reactions, as well as metal catalysts using non-noble metals? How can organic chemists work with computational scientists to accurately predict chemical, physical, and biological properties of organic molecules through reliable structure–property, structure–activity, and structure–function relationship studies? How can computational organic chemists construct a reliable program for indicating most efficient synthetic routes to organic compounds with certain structural complexity?

Of course, there are numerous challenges within the realm of organic chemistry and its branches. Creation of new chemical entities (NCEs) can only be achieved by chemists- no other science discipline can compete with chemistry in this respect. Then synthetic organic chemistry is responsible for all organic NCEs. Accordingly, both innovative and incremental advances in synthetic methods and methodologies are significant in this respect. In addition to the exploration of more selective, efficient and “greener” chemical processes, especially using metal or organic catalysts, development of highly efficient catalyst recovery and product separation technologies is critical, which has relevance to sustainability and environmental issues. Innovative synthetic methods and methodologies that enable late-stage modifications will significantly accelerate the analog design and synthesis in medicinal chemistry and drug discovery. Chemical informatics will play increasingly important role in synthetic organic and medicinal chemistry as well as organic materials chemistry. Computational analysis and design will also play critical role in medicinal chemistry, drug discovery, catalysis, supramolecular chemistry, and organic materials.

## Author contributions

The author confirms being the sole contributor of this work and approved it for publication.

### Conflict of interest statement

The author declares that the research was conducted in the absence of any commercial or financial relationships that could be construed as a potential conflict of interest.

